# A Severe Case of Overlap of Morphea and Eosinophilic Fasciitis after Burn Injuries

**DOI:** 10.1155/2024/3123953

**Published:** 2024-05-14

**Authors:** Hania Sami, Faria Sami, Shahzad Ahmed Sami, Abdulqadir J. Nashwan

**Affiliations:** ^1^Shalamar Medical and Dental College, Lahore, Pakistan; ^2^Internal Medicine, John H. Stroger Jr. Hospital of Cook County, Chicago, IL, USA; ^3^Internal Medicine, Trinity Health Oakland Campus, Pontiac, Michigan, USA; ^4^Nursing Department, Hamad Medical Corporation, Doha, Qatar

## Abstract

**Background:**

Generalized morphea is a rare fibrosing skin illness that progresses from erythematous, violet-colored skin patches to sclerotic plaques. Another uncommon immune-mediated connective tissue disease called eosinophilic fasciitis (EF) evolves to cause sclerosis and woody skin induration. The coexistence of the two is extremely rare and has a poorer prognosis. Our case report is one of the first to report burn injuries as a trigger factor for EF and generalized morphea overlap. *Case Presentation*. A 36-year-old man presented with acute onset of rapidly progressing skin thickening, tender edema, and skin contractures involving all extremities, shortly after enduring burn injuries from a gasoline explosion. Workup was remarkable for peripheral eosinophilia, hypergammaglobulinemia, and elevated C-reactive protein. Skin biopsy demonstrated sclerodermoid changes and sclerotic thickening of subcutaneous fibrous septa associated with stromal mucin, dermal perivascular, diffuse lymphoplasmacytic infiltrate with eosinophils, decreased CD34 expression, and increased factor XIIIa. He was subsequently diagnosed with an overlap of generalized morphea and eosinophilic fasciitis. The patient had only limited improvement with steroids, methotrexate, mycophenolate mofetil, and intralesional triamcinolone acetonide injections.

**Conclusion:**

Generalized morphea with concomitant EF indicates some degree of therapeutic resistance and poor prognosis with a low quality of life. Burn injuries can be a trigger factor for this overlap syndrome. Prompt identification of at-risk individuals and initiating aggressive management are necessary.

## 1. Introduction

Generalized morphea, also called “localized scleroderma,” is a rare fibrosing illness causing erythematous skin patches and plaques evolving into areas of sclerosis with postinflammatory hyperpigmentation [1]. Eosinophilic fasciitis (EF) is a connective tissue disorder characterized by pitting edema and erythema followed by woody induration due to overexpression of cytokines [2]. Overlap of these conditions is a rare and atypical presentation resulting from poorly understood pathogenic mechanisms [3]. Several trigger factors are speculated in the literature including trauma and chemical exposures. We present a unique case of this overlap triggered after enduring burn injuries from a gasoline explosion.

## 2. Case Presentation

A 36-year-old man presented with acute onset of rapidly progressive bilateral upper and lower extremity skin thickening, painful erythematous edema, and contractures for 2 months. He had suffered from second-degree burn injuries on bilateral upper extremities including the dorsal surface of the hands, the left forearm and arm, and a small area on the left shoulder from a gasoline explosion at work as a construction laborer six months before the presentation who had healed without complications within a month. He denied any new medications, other occupational or environmental exposures, trauma, and exercise. On examination, his hands were fixed in a grip position with noticeable tenderness and erythematous edema, nonconfluent patches of scarring, and induration spread unevenly throughout the extremities and peripherally on the abdomen. The elbows were unable to flex, and knees had a limited range of motion secondary to skin induration and contractures. Muscle strength evaluation was limited due to extreme rigidity and limited range of motion in bilateral extremities and fingers.

**Figure 1:**
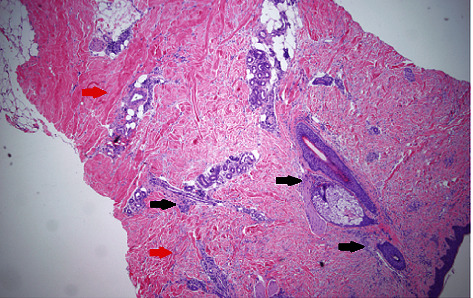
Skin biopsy with ×40 magnified view showing perivascular and periadnexal inflammatory infiltrates (black arrows) and diffuse sclerosis (red arrows) (hematoxylin and eosin stains).

**Figure 2:**
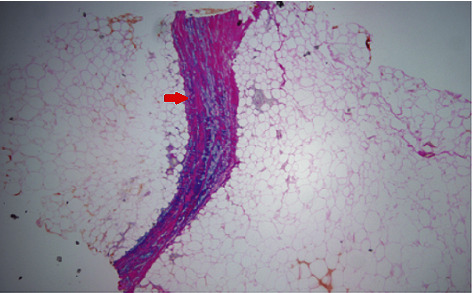
Thickened fibrous septum (red arrow) and inflammatory infiltrates in the subcutaneous layer with mucin deposition at ×40 magnification (hematoxylin and eosin stains).

**Figure 3:**
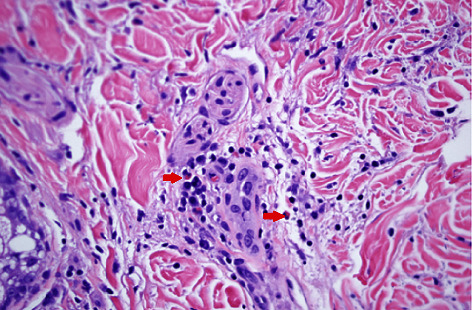
Inflammatory infiltrates with eosinophils (red arrows) dispersed in the dermis seen at ×200 magnification (hematoxylin and eosin stains).

Initial lab tests were remarkable for white cell count 9 k/*μ*L with 19% eosinophils, absolute eosinophilic count of 1.9 k/*μ*L, total protein of 8.9 g/dL, hypergammaglobulinemia 2.72 g/dL, C-reactive protein (CRP) of 1.68 mg/dL (normal <0.3 mg/dL), normal erythrocyte sedimentation rate (ESR), and negative antinuclear antibodies (ANA). Magnetic resonance imaging (MRI) of the upper extremity showed nonspecific skin thickening only with no abnormal signal changes identified. Skin biopsy revealed sclerodermic changes in the reticular dermis and sclerotic thickening of subcutaneous fibrous septa with profound stromal mucin, dermal perivascular lymphoplasmacytic infiltrate with eosinophils and increased fibroblasts, and T and plasma cells. Diffuse sclerosis, perivascular lymphocytes, and elastic fiber fragmentation were also noted. Decreased dermal and fascial CD34 expression was noted with increased factor XIIIa. These findings are seen in EF and morphea both (Figures 1–3). The patient was started on 40 mg prednisone daily and methotrexate after biopsy reported during the same admission. Methotrexate was slowly titrated up according to the treatment response in the clinic at monthly follow-up visits.

**Figure 4:**
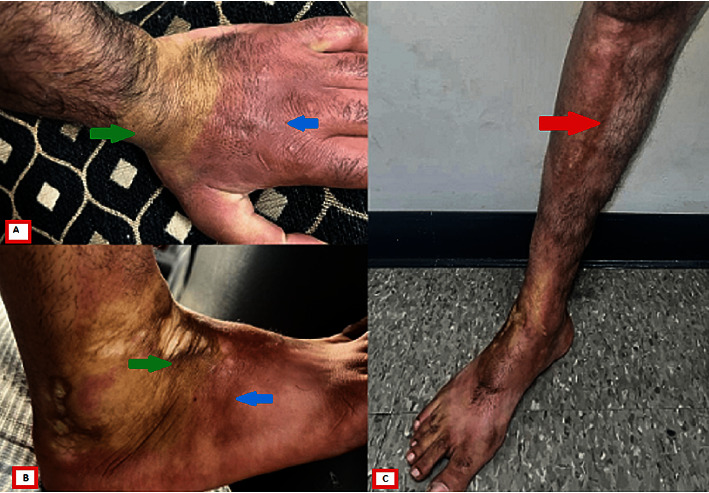
Skin findings at 1-year follow-up: (A, B) the indurated skin with painful erythematous edema (blue arrows) and nonconfluent patches of sclerosis (green arrows). (C) The indurated skin with Groove's sign on the lower extremity (red arrow).

At follow-up visits, labs showed improving but persistent eosinophilia and hypergammaglobulinemia. He gradually regained some mobility of his joints, but his improvement plateaued at the fourth month (Figure 4). Methotrexate was switched to mycophenolate mofetil 1500 mg twice daily after that with continued but very slow improvement for another year. The patient was maintained on tapering doses of steroids for 9 more months and eventually discontinued. Dermatology service started intralesional triamcinolone acetonide injections and planned to initiate ultraviolet B radiation therapy at his most recent clinic visit.

## 3. Discussion

Generalized morphea involves inflammation causing gradual sclerodermoid and atrophic changes in the skin and sometimes the underlying tissues such as fascia, muscle, and bone [4]. Several trigger factors for morphea include repetitive trauma to the involved body area, therapeutic radiation, infection, and autoimmune disorders [5]. The family history of morphea and other autoimmune illnesses is more prevalent in morphea patients than in the general population [6]. The age of presentation is bimodal, and females are more commonly affected [7].

Eosinophilic fasciitis is also a connective tissue disorder that can involve subcutaneous fascia and even deep fascial layers causing the abrupt onset of sclerosis and painful induration. Intense exercise, muscle trauma, radiotherapy, medications, and chemical exposures are some causative factors [6]. It has also been linked to autoimmune and hematological disorders, infections, and neoplasms [8]. Both sexes are equally affected, and the disease manifests between the 4 and 5th decades of life [9, 10]. Set guidelines for the diagnosis of EF are lacking, but proposed diagnostic criteria include evidence of clinical, serological, and histopathological findings and lack of evidence for other etiologies [11].

Differentiating between EF and generalized morphea can present a diagnostic challenge. EF is more likely to be acute in onset, symmetric, and has peripheral eosinophilia, which can assist in forming a diagnosis but are nonspecific findings [12]. Clinically, if systemic involvement is ruled out, generalized morphea is defined by ≥4 lesions (circumscribed or deep) in two or more anatomic locations. Diminished CD34 expression in the dermis on immunochemistry and increased factor XIIIa are noted in morphea [8]. Focal loss of expression of CD34 in the fascia is more likely in EF [13]. Imaging such as MRI, ultrasound, and positron emission tomography (PET) scan can define the lesion depth and fascial involvement. Full-thickness incisional biopsy involving the muscle is recommended [7].

There are only a handful of cases of the overlap syndrome reported. We review the literature for the overlap of generalized morphea and EF. Luo G et al. report a case of overlap of the two diagnosed on biopsy in a 45-year-old patient with long-standing skin sclerosis and also found to have IgA nephropathy [14]. Lakjiri et al. report a 45-year-old with twenty years of vitiligo who got diagnosed with this overlap syndrome [15]. Watanabe et al. also reported a case of a 60-year-old male with differential expression of CD34 in both types of lesions, discussing the origin from different mesenchymal cells and emphasizing that the two conditions are independent entities [16]. Gutierrez et al. discussed a case of a 71-year-old who presented with a one-year history of skin tightening and was found to have eosinophilia, hypergammaglobulinemia, and diminished CD34 expression on pathology, treated with intravenous immunoglobulins (IVIG) with marked improvement within months [17]. Heidary et al. reported a case of a 50-year-old lady with three months of violaceous plaques and induration, decreased CD34 expression, and absence of peripheral eosinophilia who eventually responded to treatment with methotrexate, steroids, and etanercept [18]. In this case, the biopsy was more suggestive of morphea, but based on clinical progression, the patient was diagnosed with both morphea and EF. Ergum et al. reported 4 cases of EF with three overlapping with morphea, and two of those also had lichen sclerosis [3]. They discuss a possible overlap between these fibrosing dermatological conditions from a similar inflammatory pathway. This further raises concern if burn injuries also provided a similar stimulus for our patient to trigger this overlap. The article further discusses the need for individualized treatment with adjuvant therapies such as methotrexate, hydroxychloroquine, and ultraviolet A as used for their patients with good response. More recently, Chan et al. discussed epoxy resin as a possible trigger factor for overlap of EF and morphea by discussing a case of a 24-year-old patient working as a painter presenting with acute onset of diffuse hyperpigmented sclerosis and painful edema three weeks after starting the job and progressing over seven years [19]. He had peripheral and bone marrow eosinophilia, hypergammaglobulinemia, and elevated ESR, CRP, and ANA titers. His symptoms remained refractory to multiple treatment modalities.

Treatment options include steroids, methotrexate, Ultraviolet A1/B, mycophenolate mofetil, azathioprine, and cyclosporine. It is speculated that the overlap of the two diseases portends a poorer prognosis and treatment resistance [20]. Our patient also showed therapeutic resistance with only mild improvement.

Timing and characteristics strongly indicate burn injuries as a trigger factor for our patient. To our knowledge, it is the first time this overlap secondary to burns has been reported. As EF and generalized morphea overlap is somewhat refractory to treatment, emphasis should be on early diagnosis by vigilant screening of at-risk individuals for aggressive management. There also remains a need to identify potential triggers and associations that can alter prognosis.

## Data Availability

All data generated or analyzed during this study are included in this published article. Further inquiries can be directed to the corresponding author.
